# The Presidential Address and Modern Science

**Published:** 1886-08

**Authors:** F. A. Schmitt

**Affiliations:** Schulenburg, Texas


### THE PRESIDENTIAL ADDRESS AND MODERN SCIENCE.

*Open Letter to President Becton T. S. M. A. By F. A. Schmitt, M. D., Schulenburg, Texas.*

(For Daniel's Texas Medical Journal.)

EDITOR DANIEL'S TEXAS MEDICAL JOURNAL.

It afforded me both pleasure and satisfaction, to find at least one of our State Medical Journals vindicating the good scientific standing of the Medical profession of Texas and at the same time courageous enough to resent an insult offered to science and to two of our foremost scientists.

In order to show you and an enlightened public that there are others of the profession in Texas, who keenly felt the insult offered and at the same time to criticize that portion of Dr. Becton's address which was so offending to both science and scientists, I append a verbatim copy of letter written by myself and addressed and mailed to Dr. E. P. Becton, of Sulphur Springs, Texas, immediately after reading his address in the Texas Courier Record published at Fort Worth, Texas.

Respectfully yours,

F. A. SCHMITT, M. D.

SCHULENBURG, TEXAS, June 21, 1886.

DR. E. P. BECTON, LATE PRES. T. S. M. A., SULPHUR SPRINGS:

Respected Sir: In your address before the State Medical Association at Dallas you chose to use the following language:

"But the most unkindest cut of all is, that the tendency of the profession in America is towards materialism and infidelity, etc. For this there is not the slightest foundation. [You are right, Dr., there is not.—S.]

"Now and then some *misguided* [?] member of the profession accepts the teachings of such men as Darwin and Huxley; but the great body [?] of American physicians know that these doctrines are as withering, blighting as the winter's frost, that they 'cramp the genius, freeze the vivid and glowing aspirations of the mind, and clip with unsparing hand the lofty flights of the intellect,' *that they hush the still small voice of conscience here, and damn the soul hereafter.*"

If scientific labor has such demoralizing effect, why, I ask, not stop it altogether—why not declare it a felony, punishable as such, and thereby teach these scientific fellows how to behave and to keep within the boundaries of Biblical teaching?

If you had addressed a congregation from the pulpit, your utterances would have received the hearty consent of every well-meaning Christian; the most learned scientist, as well as the humblest illiterati would have exalted over your "scriptural eloquence;" but carrying such slanderous and insulting language *to both scientists and science* into the halls of the representative scientific body of a great State, is more than the medical profession can endorse, and I for one *emphatically deny* that the medical profession of Texas is in sympathy with your views and utterances; i. e., that *scientific research and the deductions therefrom* are as withering, blighting as winter's frost, or that they *cramp* the genius, freeze the vivid and glowing aspirations of the mind, and clip with unsparing hand the lofty flights of the intellect—or that *they hush the still small voice of conscience here and damn the soul hereafter.*

I venture to assert the contrary, sir:—I take upon myself the responsibility for saying, that the lofty flights of the intellect can but be enhanced through the medium of the researches, discoveries and deductions of the very scientists you chose to name with such utter disrespect and disregard to their scientific standing and acknowledged greatness.

I further make bold to say that such doctrines as you chose to spread before the representative scientific body of our State are unworthy a man of our noble profession and of your standing as such—representing, as you have done as President of our State Society, the profession of Texas; and that such views as you hold regarding science and scripture belong, not to the nineteenth century, but to the middle ages, when science was spurned and theological sophistry held its sway.

I have not the honor of your personal acquaintance, and therefore this letter may surprise you, as also may do its more or less aggressive spirit, but as public men, their actions and shortcomings ought always to be open to fair criticism, I take advantage of this privilege to express my thoughts in the matter, and to censure where, in my estimation, censure is duly deserved.

Respectfully,

F. A. SCHMITT, M. D.

